# Correction for Piliper et al., “Clinical validation of an RSV neutralization assay and analysis of cross-sectional sera associated with 2021–2023 RSV outbreaks to investigate the immunity debt hypothesis”

**DOI:** 10.1128/spectrum.00412-25

**Published:** 2025-12-23

**Authors:** Eli A. Piliper, Jonathan C. Reed, Alexander L. Greninger

## AUTHOR CORRECTION

Volume 12, no. 12, e02115-24, 2024, https://doi.org/10.1128/spectrum.02115-24.

We discovered that calculation of titers for 10 serum samples run in the same batch on the same day failed to account for a 20-fold pre-dilution. All 10 specimens were from RSV PCR-positive individuals in the emergency department (ED), including 7 immunocompetent and 3 immunosuppressed individuals. Correction of the values for the pre-dilution leads to a 20-fold increase in the final titers for specimens from Individuals in Table S6: 06B4, 131F, 0646, 0CF5, 06E0, 029D, 11DB, 085B, 130E, and 0DEE. While comprehensively reviewing all data due to pre-dilution issue, we also found one specimen for individual 0786 from the RSV PCR-positive immunocompetent cohort that should not have passed QC and subsequently removed this specimen’s data from the analysis. The increased titers in these individuals significantly reduced the variance of RSV nAb titers in these cohorts and led to more statistically significant differences among individuals recently infected with RSV compared to the general population and individuals who later developed RSV infection, as one would hypothesize. We have updated Figure 3, Figure S1, and Table S6 to reflect the change in neutralization titer for the 10 samples and additional removed sample.

Page 1, lines 14-17 should read “Individuals recently having tested RT-qPCR for RSV had a 7.5-fold higher geometric mean neutralizing titer relative to RSV PCR negatives (*P* < 0.0001).”

Page 7-8, last paragraph, lines 7-17 should read “Neutralizing GMT of the combined cross-sectional HSV serology remnant specimen groups described above was 4.2-fold lower than the immunocompetent RSV PCR-positive individuals (two-sided *t*-test, *P* = 0.0004), 1.8-fold higher than that of RSV PCR-positive, immunosuppressed individuals (*P* = 0.17), and 1.8-fold higher than that of influenza PCR-positive, RSV PCR-negative individuals (*P* = 0.04). The future-RSV PCR-positive samples had a GMT 5.7-fold lower than PCR positive individuals without immunosuppression (*P* = 0.00017) and 1.3-fold higher than the PCR-positive, immunosuppressed individuals (*P* = 0.56). Immunocompetent RSV PCR-positive individuals had a GMT 7.5-fold higher than that of RSV PCR-positive immunosuppressed individuals (*P* = 0.0008) and 7.5-fold higher than influenza virus PCR-positive, RSV PCR-negative individuals (*P* < 0.0001).”

Page 9, last paragraph, lines 12-16 should read “For example, we noticed that GMT across sera of RSV PCR-positive individuals (without immunosuppression) sourced from one hospital trended slightly lower compared to those from the two other hospitals used in the study by 1.81-fold and 0.89-fold, respectively (Figure S1), although this difference did not reach statistical significance.”

While reviewing these data, we found several small calculation errors in the Tables and Supplemental Tables, which are listed below. We have also hosted the data used in the paper, and the R scripts used in the analysis, on GitHub. The repository can be accessed at https://github.com/greninger-lab/rsv_neut_paper.

Page 3, Table 2 was submitted with Sample IDs mistakenly swapped between the two standards and showed arithmetic means with an unrounded IU/mL conversion factor instead of geometric means with a conversion factor rounded to two decimal places, which affected the values by 0.5% and 2.6%. The corrected Table 2 should appear as shown below.

**TABLE 2:** Comparison of neutralization values obtained by the RSV FRNT assay using RSV B WV/14617/85 as the challenge virus with published estimates

Sample	N	IU/mL RSV FRNT^*a*^^,^^*c*^	IU/mL reference*^b,c^*	Ratio of IU/mL RSV FRNT / reference
NR-21973	6	13,825	12,059	1.1
NR-4022	6	1,137	1,005	1.1

^*a*^ NIBSC 16/284 standard was tested six times, resulting in a geometric mean ND50 of 2,127. IU/mL values were obtained by multiplying ND50 measurements by the conversion factor 0.94. McDonald et al. (34) reported a mean conversion factor across all assays of 1.1 but did not report a per-assay conversion factor (34).

^*b*^ Reference values obtained from McDonald et al. (34).

^*c*^ Data represent the GMT.

Page 5, Table 3 was submitted with arithmetic means for IU/mL measurements instead of geometric means, which affected the values by about 1-2%. Geometric coefficient of variance (GCV) values for the ND80 subsection of the table were mistakenly calculated with a base-2 exponential of the standard deviation instead of the base-e exponential of the standard deviation, which affected absolute GCV values by 2-5%. The corrected Table 3 should appear as shown below. Page 5, Paragraph 2, lines 6-8: Results discussing Table 3 should read: “Linearity panel specimen within-lab GCV values ranged from 5.5% to 27.8% and 18.7% to 24.9% for ND50 and ND80 measurements, respectively (Figure 1C; Table 3).” These changes in values make the assay more precise and result in improved performance.

**TABLE 3:** Intra-assay, inter-assay, and within-lab imprecision of ND50 and ND80 measurements of linearity panel specimens*^b^*

	Panel member	Expected measurement (IU/mL)	*n* ^*a*^	Geometric mean (IU/mL)	Intra-assay component		Inter-assay component		Within-lab	
					Geometric SD	%GCV	Geometric SD	%GCV	Geometric SD	%GCV
ND50	2	1,386	6	983	1.14	12.9	1.20	18.0	1.25	22.3
	3	346	6	272	1.11	10.9	1.24	21.8	1.27	24.5
	4	87	6	73	1.16	14.6	1.26	23.5	1.31	27.8
	5	22	6	20	1.07	6.9	0.00	0.0	1.06	5.5
ND80	2	1,263	6	1108	1.12	10.9	1.25	22.2	1.28	24.9
	3	315	6	289	1.09	8.9	1.21	19.5	1.24	21.5
	4	79	6	75	1.09	8.7	1.18	16.4	1.20	18.7
	5	20	6	19	1.28	25.2	0.00	0.0	1.25	22.8

^*a*^ Total number of replicates tested.

^*b*^ Total number of replicates tested. All neutralizing titer values were averaged. Geometric SD and CV were calculated. Negative variance estimates from ANOVA analysis were reported as a GSD of zero.

Page 6, Table 4: We also included GCV values that were not correctly adjusted for using log_2_-transformed titers with a natural log. We also derived slightly different (<0.5%) geometric mean IU/mL values. The correct Table 4 is shown below with corrected GCV and geometric mean measurements. Page 5, paragraph 2, lines 8-12: Results discussing Table 4 should read “Remnant rubella specimens had within-lab GCV values ranging from 3.2 to 15.5% and 3.2 to 17.6% for ND50 and ND80 measurements, respectively (Figure 1D; Table 4).” These changes in values make the assay more precise and result in improved performance.

**TABLE 4:** Intra-assay, inter-assay, and within-lab imprecision of ND50 and ND80 measurements of remnant clinical specimens*^b^*

Panel member	*n* ^*a*^	IU/mL from ND50			IU/mL from ND80		
		Geometric mean	Geometric SD	%GCV (within lab)	Geometric mean	Geometric SD	%GCV (within lab)
R-01	4	510	1.06	6.0	600	1.06	6.0
R-02	4	145	1.17	15.5	143	1.19	17.6
R-08	4	835	1.09	8.7	983	1.09	8.7
R-12	4	1,190	1.05	5.1	1,345	1.10	9.3
R-14	4	1,367	1.03	3.2	1,609	1.03	3.2

^*a*^ Total number of replicates tested.

^*b*^ All neutralizing titer values were averaged, and geometric SD and total within-lab imprecision was estimated.

Page 6, Table 5: We also derived slightly different (<0.5%) geometric mean IU/mL values for RSV-B standards, which does not affect the overall performance of the assay. The corrected Table 5 should appear as shown below.

**TABLE 5:** Intra-assay, inter-assay, and within-lab imprecision of ND50 and ND80 measurements of contrived specimens using RSV B WV/14617/85 as challenge virus^*b*^

Sample	*n^a^*	Geometric mean (IU/mL)	Intra-assay component		Inter-assay component		Within-lab	
			Geometric SD	%GCV	Geometric SD	%GCV	Geometric SD	%GCV
NIBSC 16/284	6	2,000	1.28	24.8	1.11	10.6	1.31	27.1
NR-21973	6	13,825	1.26	23.5	1.02	2.1	1.26	23.6
NR-4022	6	1,137	1.05	5.2	1.11	10.5	1.12	11.8

^*a*^ Total number of replicates tested.

^*b*^ Three reference sera were tested in duplicate over 3 days with RSV B WV/14617/85 in place of RSV A2. All neutralizing titer values were averaged. Geometric SD and GCV were calculated.

Page 7, Fig. 2: The solid lines were detailed as showing the geometric mean, but they were instead showing the arithmetic mean. The figure has been updated such that the solid lines now show the geometric mean.

Page 13, Table S1: The RSV-B ND50 and ND80 values were calculated with arithmetic means instead of geometric means, which affected the ND50 by ~3% and ND80 by 0.4%. The updated table shows correct use of the geometric mean results in an ND50 RSV-B conversion factor to IU/mL of 0.94, rather than 0.91, and an ND80 conversion factor of 3.40, rather than 3.39. Page 2, last paragraph, lines 10-12 should read “The conversion factors for RSV A2 and RSV B WV/14617/85 FRNT ND50 units to IU/mL were 0.37 and 0.94, while the ND80 conversion factors were 1.32 and 3.40, respectively (Table S1).” Page 9, Paragraph 2, last two lines should read “Our RSV B conversion factor from ND50 to IU/mL was 0.94, similar to the average conversion factor of 1.1 calculated from NIBSC (34).” The correct Table S1 should appear as shown in this correction

Page 13, Table S5 and S6: As noted above, the line-item titer values in Table S6 values are updated. The correct Table S6 should appear as shown in this correction.

A revised version of the manuscript that corrects these errors is being published simultaneously (https://doi.org/10.1128/spectrum.01739-25).

